# *NRG1* fusion-positive solid tumors: clinical detection, genomic landscape, and real-world data in pancreatic cancer

**DOI:** 10.1093/jnci/djaf361

**Published:** 2025-12-13

**Authors:** Alison M Schram, Soo-Ryum Yang, Genessa Kahn, Rogelio Velasco, Matteo Repetto, Richard Kinh Gian Do, Sara DiNapoli, Purvil Sukhadia, Megan Troxel, Kerem Ozcan, Zeynep Tarcan, Marc Ladanyi, James J Harding, Alexander Drilon, Olca Basturk, Eileen M O’Reilly

**Affiliations:** Department of Medicine, Memorial Sloan Kettering Cancer Center, New York, NY 10065, USA; Department of Pathology and Laboratory Medicine, Memorial Sloan Kettering Cancer Center, New York, NY 10065, USA; Department of Medicine, Memorial Sloan Kettering Cancer Center, New York, NY 10065, USA; Hunter College, New York, NY 10065, USA; Department of Thoracic Oncology, Lung Center of the Philippines, Quezon City 1100, Philippines; Department of Medicine, Memorial Sloan Kettering Cancer Center, New York, NY 10065, USA; Department of Radiology, Memorial Sloan Kettering Cancer Center, New York, NY 10065, USA; Department of Pathology and Laboratory Medicine, Memorial Sloan Kettering Cancer Center, New York, NY 10065, USA; Department of Pathology and Laboratory Medicine, Memorial Sloan Kettering Cancer Center, New York, NY 10065, USA; Department of Medicine, Memorial Sloan Kettering Cancer Center, New York, NY 10065, USA; Department of Pathology and Laboratory Medicine, Memorial Sloan Kettering Cancer Center, New York, NY 10065, USA; Department of Pathology and Laboratory Medicine, Memorial Sloan Kettering Cancer Center, New York, NY 10065, USA; Department of Pathology and Laboratory Medicine, Memorial Sloan Kettering Cancer Center, New York, NY 10065, USA; Department of Medicine, Memorial Sloan Kettering Cancer Center, New York, NY 10065, USA; Department of Medicine, Memorial Sloan Kettering Cancer Center, New York, NY 10065, USA; Department of Pathology and Laboratory Medicine, Memorial Sloan Kettering Cancer Center, New York, NY 10065, USA; Department of Medicine, Memorial Sloan Kettering Cancer Center, New York, NY 10065, USA

## Abstract

**Background:**

*NRG1* fusions are unique oncogenic drivers that activate the HER3/HER2/PI3K pathway. The US Food and Drug Administration granted accelerated approval to a HER2/HER3 antibody, zenocutuzumab, for treatment of *NRG1* fusion-positive non–small cell lung cancer and pancreatic ductal adenocarcinoma (PDAC). The optimal detection methods and clinicopathological features of patients with *NRG1* fusion-positive cancer have not been systematically studied. Here, we review *NRG1* fusion-positive cancer and focus on outcomes in PDAC.

**Methods:**

Patients with *NRG1* fusion-positive disease at Memorial Sloan Kettering Cancer Center were identified using institutional databases. Clinicopathological data were extracted from medical records. *NRG1* fusion-positive PDAC cases underwent review of radiology, pathology, treatment data, and assessment of progression-free and overall survival.

**Results:**

Of 76 531 patients, 48 *NRG1* fusion-positive cases were identified. The most common tumor types were lung (60%), PDAC (21%), and breast (10%). Approximately half (46%) of these patients received HER2- and/or HER3-directed therapy. Patients were identified by RNA (*n* = 34), DNA (*n* = 11), or both (*n* = 3). RNA was superior to DNA for fusion identification. Twenty-one fusion partners were detected, most commonly CD74 (40%) and ATP1B1 (10%). Lung cancers were otherwise driver negative, and PDAC cases were *KRAS* wild type. *NRG1* fusion-positive PDAC exhibited distinct histopathological and clinical features. Median patient age was 48.5 years, median progression-free survival on first-line chemotherapy was 12.6 months (*n* = 7; 95% confidence interval [CI] = 2.9 to not reached), and median overall survival from diagnosis was 39.6 months (*n* = 9; 95% CI = 23.2 to not reached).

**Conclusions:**

NRG1 fusions are a newly described, clinically actionable target in solid tumors. We report the landscape of NRG1 fusion-positive cancers and highlight the importance of RNA testing. NRG1 fusion-positive PDAC is enriched in younger patients with *KRAS* wild-type disease and has unique biology.

## Introduction

Gene fusions occur across solid tumors and typically drive oncogenesis through constitutive activation of tyrosine kinases, increased downstream signaling, and uncontrolled tumor growth. Small molecule inhibitors targeting fusions involving ALK, ROS1, RET, and TRK have demonstrated remarkable activity across several cancer types, transforming the treatment paradigm and improving prognosis for patients harboring these alterations.^1^^,^^2^

Neuregulin 1 (NRG1) is a ligand that binds to HER3, promoting HER2-HER3 heterodimerization and PI3K/AKT/mTOR signaling activation.^3-5^ Genomic rearrangements involving *NRG1* fusion positivity have been identified in a variety of solid tumor types, with a prevalence less than 1% overall, and appear to be enriched in *KRAS* wild-type pancreatic ductal adenocarcinoma (PDAC) and driver-negative non–small cell lung cancer (NSCLC).^6-9^ Although numerous upstream fusion partners have been described, all functional fusions contain the EGF-like domain of *NRG1*, resulting in autocrine and paracrine signaling via HER2-HER3 heterodimerization.^6^^,^^7^^,^^10^ Consequently, these NRG1 ligand fusions result in increased cellular proliferation, cell migration, and tumor growth.^11-15^ NRG1 fusions therefore represent a unique biology compared with fusions in the receptor tyrosine kinases ALK, ROS1, RET, and TRK that result in ligand-independent activation.

The eNRGy1 global lung cancer registry evaluated the natural history and efficacy of anticancer therapy in *NRG1* fusion-positive NSCLC. Patients were found to respond poorly to standard-of-care treatment, with overall response rates of 20% or less and median progression-free survival (PFS) rates less than 6 months on chemotherapy, immunotherapy, and chemoimmunotherapy.^10^

Targeting HER2/HER3 signaling is a promising therapeutic approach for patients with *NRG1* fusion-positive tumors. A phase 2 global clinical trial (A Study of Zenocutuzumab (MCLA-128) in Patients With Solid Tumors Harboring an NRG1 Fusion [eNRGy], ClinicalTrials.gov identifier NCT02912949) is exploring the HER2/HER3 bispecific antibody zenocutuzumab in *NRG1* fusion-positive unresectable or metastatic solid tumors. Initial data are encouraging, with durable responses and a favorable safety profile. These data were particularly robust in patients with NSCLC and PDAC. Across solid tumors, the overall response rate was 30%, with a median duration of response of 11.1 months; in NSCLC, the overall response rate was 29%, with a median duration of response of 12.7 months; and in PDAC, the overall response rate was 42%, with a median duration of response of 7.4 months. Based on these results, the US Food and Drug Administration granted accelerated approval to zenocutuzumab for the treatment of *NRG1* fusion-positive NSCLC and PDAC.^16^ Preliminary efficacy targeting *NRG1* fusion-positive cancer has also been reported with the HER3 monoclonal antibodies GSK2849330 and seribantumab^7^^,^^17^ and the pan-HER inhibitor afatinib.^10^^,^^18^

PDAC is an aggressive disease with limited treatment options after progression on first-line therapy. It is unknown whether *NRG1* fusion-positive PDAC has a unique biology. We explored the clinicopathological characteristics of patients with *NRG1* fusion-positive cancer at Memorial Sloan Kettering Cancer Center (MSK), with a focus on real-world outcomes in PDAC.

## Methods

### Study design and participants

This was a retrospective, single-institution, noninterventional study. We included patients with *NRG1* fusion-positive solid tumors detected using tumor next-generation sequencing from July 2014 to July 2024. Patients were identified using an automated genotype-driven cohort management system and through clinical practice.^19^

Clinicopathological data, including sex, age, smoking status, age at diagnosis, location of tumor, and histopathological features, were manually extracted from medical records. When available, sequencing reads were reviewed to identify precise fusion breakpoints. The PDAC cases underwent additional pathology and radiology review of histopathology slides and computed tomography scans, respectively. Treatment data, including date of diagnosis, treatments received, dates of first and last cycles of treatment, and survival status (data cutoff, July 1, 2024), were manually abstracted.

The study was conducted in accordance with the Declaration of Helsinki and approved by the MSK Institutional Review Board.

### Fusion detection

Diagnostic methods included DNA-based (MSK-IMPACT) or RNA-based (MSK-Fusion) targeted next-generation sequencing assays. MSK-IMPACT is a hybridization capture-based next-generation sequencing assay that assesses the coding regions and selected promoter and intronic regions of 341-505 cancer genes (increased with each version) for mutations, amplifications, deletions, select structural rearrangements, and microsatellite status against a patient’s matched normal.^20-22^ However, MSK-IMPACT does not include NRG1 introns because of their size. MSK-Fusion is a custom, targeted, RNA-based panel that uses Archer Anchored Multiplex PCR technology and next-generation sequencing to detect gene fusions in 129 genes (including *NRG1*) known to be involved in chromosomal rearrangements.^20^^,^^23-25^ These assays were validated by the MSK Molecular Diagnostic Service and approved for clinical use by the New York State Department of Health Clinical Laboratory Evaluation Program. They were used alone or as part of sequential testing (eg, DNA-based next-generation sequencing first and RNA-based sequencing second). Next-generation sequencing reads were reviewed to confirm that detected *NRG1* fusions were in frame and retained the EGF-like domain (NM_013962) as a downstream fusion partner, which is critical for HER3 binding and oncogenic signaling. *NRG1* fusions that did not meet these criteria were considered structural variants of unknown significance.

### Outcomes

Clinical endpoints included PFS (start of therapy to cancer progression by Response Evaluation Criteria in Solid Tumors [RECIST], version 1.1, or death), real-world PFS (start of therapy to cancer progression by clinician assessment or death), time to treatment discontinuation (initiation of first therapy to 2 weeks after last administration or death), time to next treatment (initiation of first therapy to initiation of second-line therapy or death), and overall survival (diagnosis to death). Censoring was performed on the date patients were last known alive and receiving treatment.^26^

### Statistical analysis

Categorical variables were reported as frequencies and proportions; continuous variables were reported as median (range). The Kaplan-Meier method with R, version 4.3.1, software were used for survival analyses and 95% confidence intervals (CIs), with surviving patients censored at the date of last follow-up. Stata, version 16, software (StataCorp LP) was used for statistical analysis.

## Results

### Molecular testing

**Figure 1.**
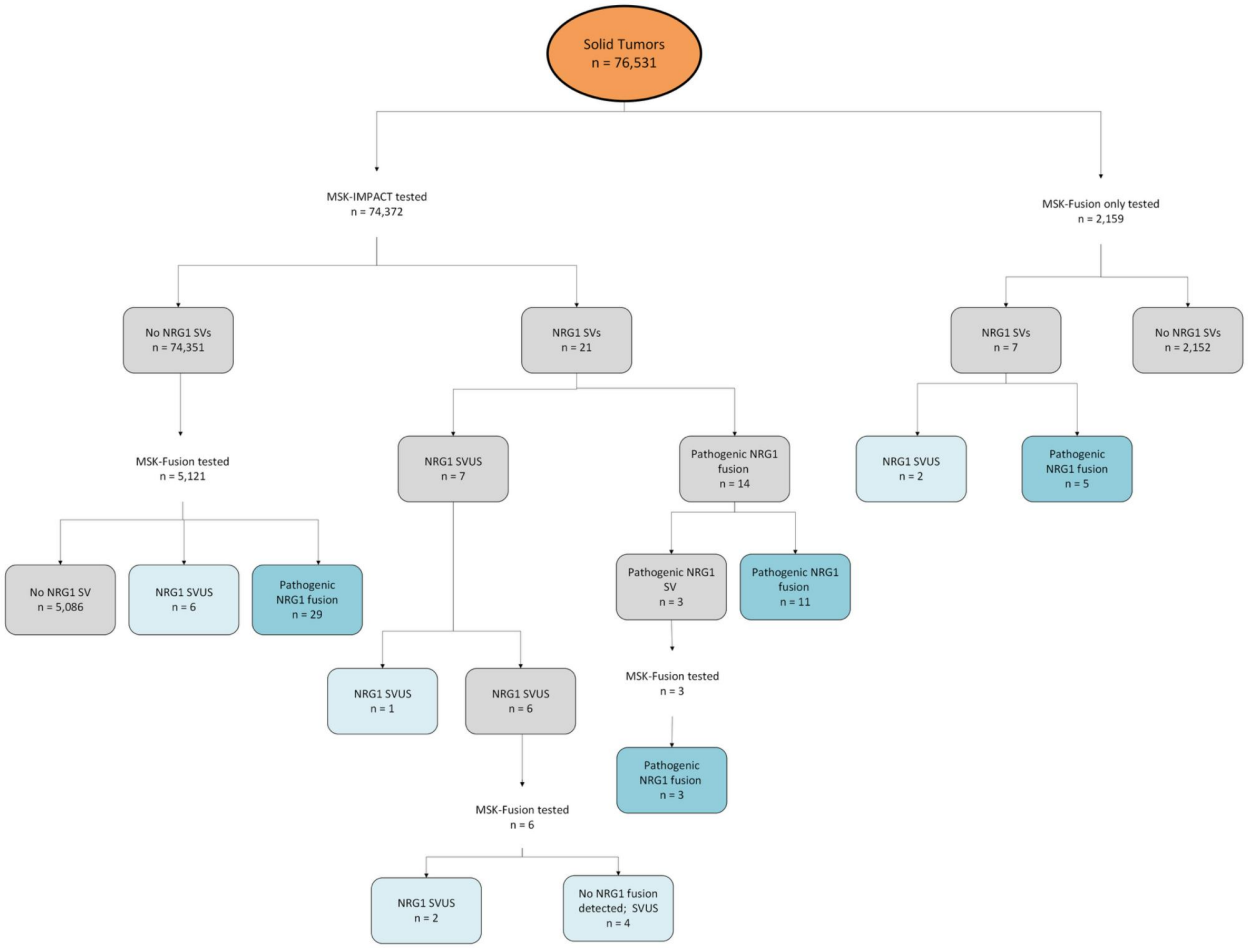
Schema of *NRG1* fusion detection at Memorial Sloan Kettering Cancer Center. This flow diagram illustrates the number of cases with *NRG1* structural variants and pathogenic fusions detected by DNA-based MSK-IMPACT testing, RNA-based MSK-Fusion testing, or both. **Dark blue boxes** represent the final count of pathogenic *NRG1* fusions, and **light blue boxes** represent the final count of *NRG1* structural variants of unknown significance.

**Figure 2.**
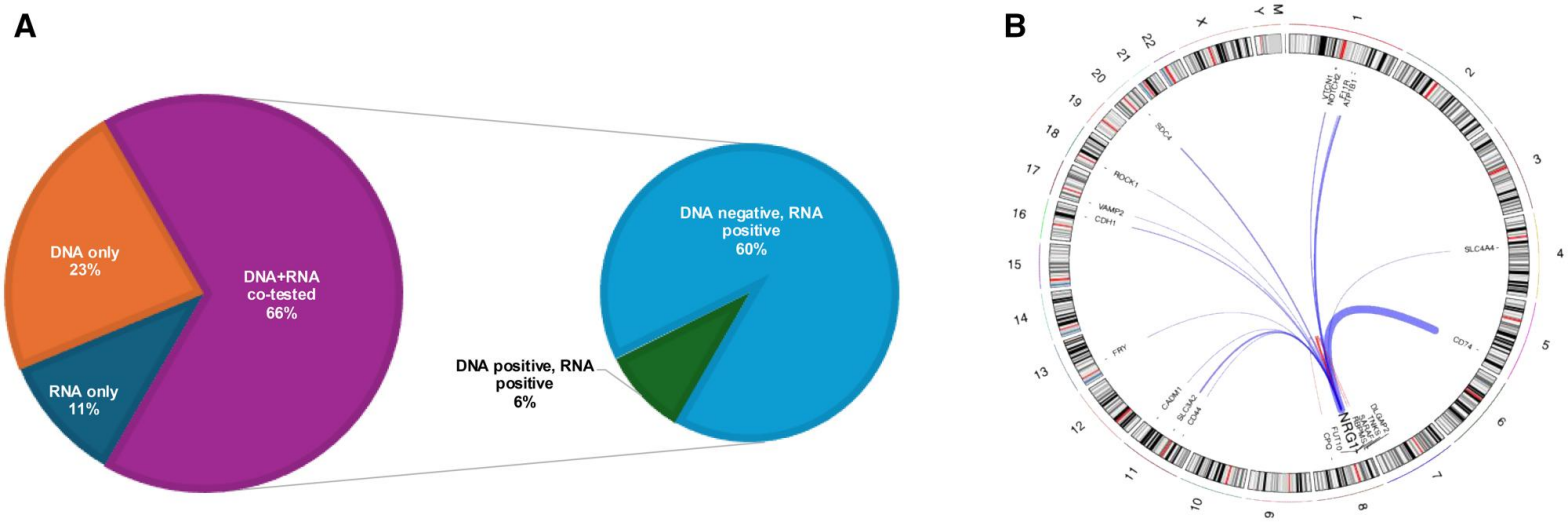
*NRG1* fusion-positive fusion detection and upstream partners. **A**) Representation of fusion detection on RNA, DNA, or both. **B**) Circos plot showing fusion events with the corresponding fusion gene partner.

Of 76 531 patients, 63 were found to have *NRG1* fusions or structural rearrangements. Forty-eight patients had pathogenic *NRG1* fusions (in frame, with retention of the EGF-like domain) (Table 1), and 15 had *NRG1* structural variants of unknown significance (Figure 1). Pathogenic *NRG1* fusions were identified using single-assay, RNA-based next-generation sequencing (*n* = 5 [10%]); single-assay, DNA-based next-generation sequencing (*n* = 11 [23%]); and sequential DNA/RNA-based next-generation sequencing testing (*n* = 32 [66%]) (Table 1; Figure 2, A). Of 32 co-tested cases, 29 (91%) were detected by RNA but not DNA. In total, 34 (71%) of patients were identified on RNA-based next-generation sequencing, 11 (23%) on DNA-based next-generation sequencing, and 3 (6%) on both.

**Table 1.** Characteristics of patients with pathogenic *NRG1* fusions.

Characteristic	Value (*n* = 48)
Age at diagnosis, median (Range), y (diagnosis of tumor containing NRG1 fusion)	61 (9-83)
Sex, No. (%)	
Female	27 (56)
Male	21 (44)
Race, No. (%)	
Asian–Far East or Indian subcontinent	7 (15)
Black or African American	2 (4)
Native Hawaiian or Pacific Islander	1 (2)
White	33 (69)
Unknown	5 (10)
Smoking status, No. (%)	
Former or current smoker	17 (35)
Never smoker	28 (58)
Unknown	3 (6)
Cancer type,^a^ No. (%)	
Lung adenocarcinoma	29 (60)
Pancreatic cancer	10 (21)
Breast cancer	5 (10)
Estrogen receptor/progesterone receptor positive, HER2 negative	4 (8)
Triple-negative breast cancer	1 (2)
Hepatobiliary	3 (6)
Intrahepatic cholangiocarcinoma	2 (4)
Gallbladder adenocarcinoma	1 (2)
Cellular neurothekeoma	1 (2)
Stage at diagnosis, No. (%)	
Localized, I-III	22 (46)
I	8 (17)
II	7 **(**15**)**
III	7 (15)
Metastatic, IV	25 (52)
Unknown	1 (2)
HER3-targeted with or without HER2-targeted therapy, No. (%)	
HER2/HER3 bispecific antibody	18 (38)
Pan-HER tyrosine kinase inhibitor	4 (8)
HER3 monoclonal antibody	3 (6)
HER2 antibody-drug conjugate	2 (4)
None	26 (54)
Fusion detection method, No. (%)	
RNA-based next-generation sequencing alone	34 (71)
DNA-based next-generation sequencing alone	11 (23)
Both RNA-based and DNA-based next-generation sequencing	3 (6)
Fusion partner, No. (%)	
* CD74*	19 (40)
* ATP1B1*	5 (10)
* SLC3A2*	4 (8)
* SDC4*	3 (6)
* CDH1*	2 (4)
Other^b^	15 (31)

a Additional tumor types were noted in patients with structural variants/fusions of unknown significance, including colorectal cancer, sarcoma, gland carcinoma, and prostate cancer.

b *SLCA4, CADM1, CD44, CPQ, F11R, FRY, FUT10, NOTCH2, RBPMS, ROCK1, SARAF, TNKS, VAMP2, VTCN1*, and *DLGAP2* (all *n* = 1).

### Patient demographics

Most patients with pathogenic *NRG1* fusions were female (56%), and median age at diagnosis was 61 years (range = 9-83 years). Most were White (69%) or Asian (15%). Patients were more often nonsmokers (58%), and the majority had stage IV disease at diagnosis (52%). The most common tumor type was lung cancer (60%). In all 29 patients with lung cancer, pathology was consistent with lung adenocarcinoma with or without mucinous differentiation. Other tumor types included PDAC (21%), breast cancer (10%), hepatobiliary cancer (6%), and cellular neurothekeoma (*n* = 1 [2%]). Notably, patients with *NRG1* structural variants of unknown significance had a range of tumor types, including colorectal cancer, sarcoma, salivary gland cancer, and prostate cancer. Approximately half (46%) of patients with presumed pathogenic *NRG1* fusions were treated with HER3-targeted with or without HER2-targeted therapy.

Of the 10 patients with pancreatic cancer, 7 were male and 3 were female (Table 2). All patients had PDAC. One patient reported exposure to Agent Orange. The median age at diagnosis was 48.5 years (range = 25-66 years). Most were never smokers with stage IV disease (*n* = 8) at diagnosis. The most common sites of metastasis were liver and lymph nodes. Two of 10 patients presented with localized disease. The first underwent surgical resection, declined adjuvant treatment, and developed progressive disease. The second underwent surgery but developed liver metastasis during adjuvant therapy.

**Table 2.** Characteristics of patients with *NRG1* fusion-positive pancreatic adenocarcinoma.

Characteristic	Value (*n* = 10)
Sex, No. (%)	
Male	7 (70)
Female	3 (30)
Age at fusion diagnosis, median (range), y	48.5 (25-66)
Ethnicity, No. (%)	
White	10 (100)
Smoking status, No. (%)	
Never	8 (80)
Former	2 (22)
Current	0 (0)
Stage at diagnosis, No. (%)	
I	1 (10)
II	1 (10)
III	0 (0)
IV	8 (80)
Pancreatic lesion location, No. (%)	
Head	4 (40)
Body	1 (20)
Tail	1 (10)
Overlapping	4 (40)
Site of metastasis,^a^ No. (%)	
Liver	8 (80)
Lymph node	8 (80)
Lung	4 (40)
Intestine	1 (10)
Bone	1 (10)
Peritoneum	1 (10)
Chemotherapy regimen (when received in early stage), No. (%)	
Gemcitabine + capecitabine	1 (10)
FOLFIRINOX	1 (10)
Not applicable	8 (80)
Systemic therapy regimens (metastatic first-line), No. (%)	
FOLFIRINOX/modified FOLFIRINOX	6 (60)
FOLFIRI	1 (10)
Gemcitabine, docetaxel, and capecitabine	1 (10)
Zenocutuzumab	1 (10)
Other^b^	1 (10)
HER3-targeted with or without HER2-targeted therapy, No. (%)	
Zenocutuzumab	6 (60)
HER3 monoclonal antibody	1 (10)
Afatinib	1 (10)
None	4 (40)
Fusion detection method, No. (%)	
RNA-based next-generation sequencing alone	10 (100)
DNA-based next-generation sequencing alone	0 (0)
Both RNA-based and DNA-based next-generation sequencing	0 (0)
Fusion partner, No. (%)	
* ATP1B1*	5 (50)
* ROCK1*	1 (10)
* CDH1*	1 (10)
* CD44*	1 (10)
* SLC4A4*	1 (10)
* NOTCH2*	1 (10)

Abbreviations: FOLFIRI = folinic acid, fluorouracil, and irinotecan; FOLFIRINOX = leucovorin calcium, fluorouracil, irinotecan hydrochloride, and oxaliplatin.

a Patients may have had more than 1 site of metastasis.

b Gemcitabine and nab-paclitaxel-based clinical trial.

### Molecular profile

**Figure 3.**
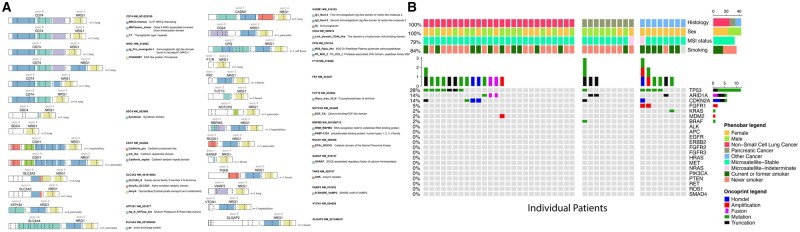
Detailed genomics of patients with *NRG1* fusion-positive solid tumors. **A**) Fusion breakpoint details for patients with *NRG1* fusion-positive cancer. **B**) Oncoprint depicting somatic alterations (mutations, copy number changes, and fusions) in patients with *NRG1* fusion-positive solid tumors with MSK-IMPACT results available.

Twenty-one unique fusion partners were detected, most commonly *CD74* (40%), *ATP1B1* (10%), *SLC3A2* (8%), *SDC4* (6%), and *CDH1* (4%) (Table 1; Figure 2, B). *NRG1* was the 3ʹ partner, and all fusions involved *NRG1* breakpoints in exons 2 through 4 (NM_013962) (Figure 3, A). All lung cancers were otherwise driver negative. No tumor had microsatellite instability or high tumor mutation burden (Figure 3, B). Germline *ATM* and *BRCA2* variations were detected in 1 patient each.

**Figure 4.**
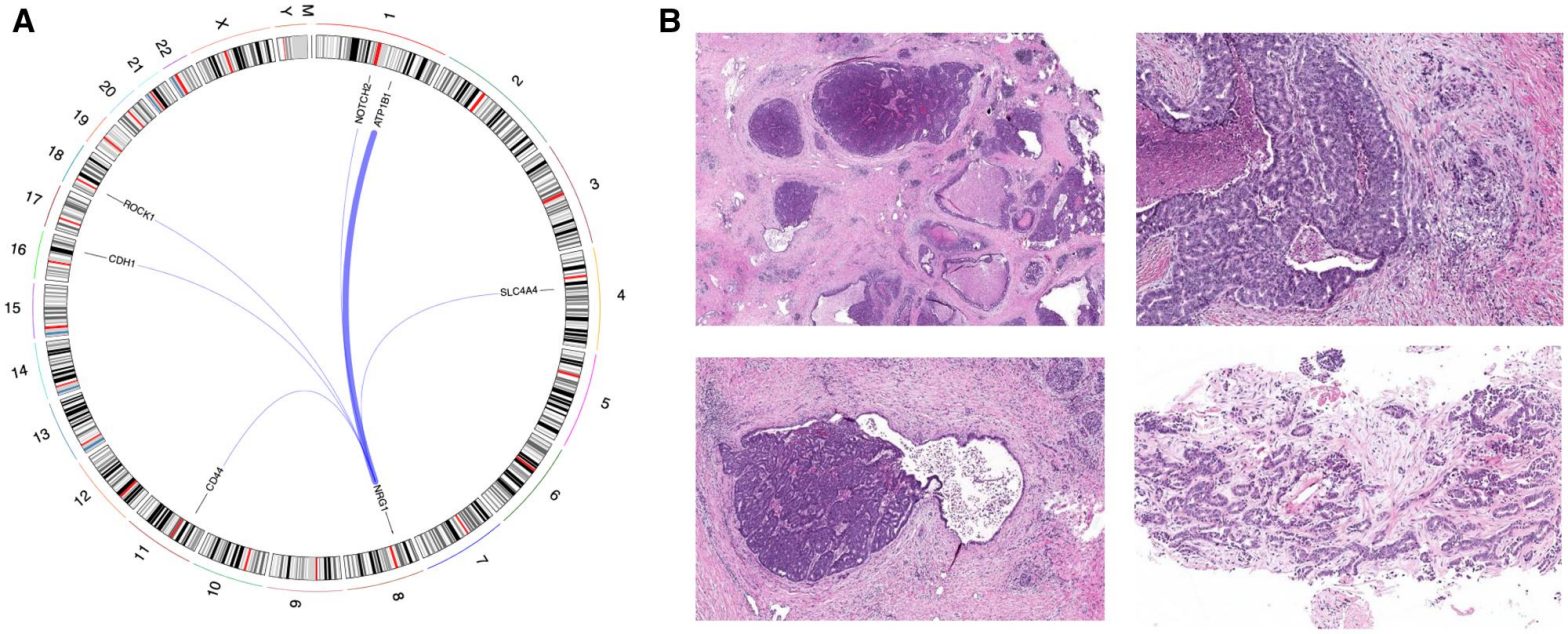
Detailed genomics and pathology of patients with *NRG1* fusion-positive pancreatic ductal adenocarcinoma. **A**) Circos plot showing fusion events with the corresponding fusion gene partner. **B**) Microscopically by H&E stain, the tumors revealed circumscribed nodules, some with comedo-type necrosis (upper left, 20x), composed of back-to-back tubular glands (upper right, 100x). Continuity of the neoplastic epithelium with benign ductal epithelium identified in some these nodules (lower left, 40x). The invasive component was characterized by thin strands of tumor cells or individual malignant glands extending away from the edges of the nodules into the stroma (upper and lower right, 100x and 20x respectively).

All 10 patients with PDAC had DNA-based and RNA-based testing performed. In all cases, the *NRG1* fusion was not detected on DNA but was identified on RNA. Fusion gene partners were *ATP1B1* (*n* = 5), *ROCK1* (*n* = 1), *CDH1* (*n* = 1), *CD44* (*n* = 1), *SLC4A4* (*n* = 1), and *NOTCH2* (*n* = 1) (Figure 4, A). All cases were *KRAS* wild type. One patient had a concurrent *BRAF* exon 12 in-frame deletion (Figure 3, B). This class 2 *BRAF* alteration has been reported in 2.3% to 7.1% of *KRAS* wild-type PDAC cases and causes *RAS*-independent *RAF* dimerization and activation of the mitogen-activated protein kinase pathway.^27^ One patient had a germline *BRCA2* mutation.

### Pathological features in PDAC

Eight of the 10 cases were diagnosed on biopsy as adenocarcinoma in the pancreas (*n* = 2) or liver (*n* = 6). One pancreatic case subsequently underwent pancreaticoduodenectomy, revealing an intraductal tubulopapillary neoplasm (ITPN) with invasive carcinoma. Among the 6 liver cases, 3 were found to harbor morphologically similar adenocarcinoma on follow-up pancreatic biopsies. Another case proceeded directly to pancreaticoduodenectomy and was diagnosed as ITPN with associated invasive carcinoma.^28-30^ The final case was initially diagnosed on liver biopsy as an atypical glandular lesion, favoring atypical bile duct adenoma, but subsequent clinical workup led to a diagnosis of PDAC, prompting reclassification of the liver lesion as metastatic PDAC.

Across all cases, the invasive carcinoma component demonstrated prominent cellularity and sparse stroma, in contrast to the abundant desmoplastic stroma typically observed in conventional PDAC. Tumor cells were cuboidal, with modest amounts of eosinophilic to amphophilic cytoplasm and round to oval, atypical nuclei. Intracytoplasmic mucin was markedly reduced (Figure 4, B). In 1 liver biopsy, these distinctive histological features were explicitly highlighted in the original pathology report, raising concern for invasive adenocarcinoma arising from an ITPN.

Review of the tumor microenvironment using hematoxylin-eosin–stained slides revealed that all tumors but 1 were associated with relatively dense fibroblastic stroma and sparse lymphocytic infiltration. Only a single tumor showed less fibrosis and more prominent lymphocytic presence. Overall, the tumor microenvironment observed in *NRG1* fusion-positive cancers was consistent with that seen in conventional PDAC.

### Radiographic findings in PDAC

Eight of 10 patients had computed tomography imaging available for review. Five patients had infiltrative pancreatic primary tumors with vascular involvement, 3 metastatic to the liver (Figure S1) and 2 with nodal metastases. One patient had a small pancreatic tail tumor with extensive metastatic disease to the liver and lung. Two other patients had post-Whipple scans, both with metastasis to liver and 1 with additional metastatic disease to lung, peritoneum, and mesenteric nodes. Overall, these imaging findings were considered typical for patients with advanced pancreatic cancer, with no distinct radiographic features unique to *NRG1* fusion-positive cancers.

### Treatment profile and outcomes in PDAC

In the metastatic setting, leucovorin calcium, fluorouracil, irinotecan hydrochloride, and oxaliplatin (FOLFIRINOX) or modified FOLFIRINOX was the most used first-line therapy (60%). Among patients with evaluable data, median real-world PFS on first-line chemotherapy was 12.6 months (95% CI = 2.9 to not reached) (Figure S2A); median time to treatment discontinuation (*n* = 7) was 10.2 months (95% CI = 3.0 to not reached) (Figure S2B), and median time to next treatment (*n* = 4) was 13.1 months (95% CI = 2.5 to not reached). Median overall survival from diagnosis was 39.6 months (95% CI = 23.2 to not reached) (Figure S2C) and from diagnosis of metastatic disease was 37.6 months (*n* = 9; 95% CI = 23.2 to not reached) (Figure S2D).

Six of 10 patients were treated with HER3-targeted therapy with or without HER2-targeted therapy (Table 2). All 6 patients received zenocutuzumab on single-patient protocols or the eNRGy study. Five patients treated with zenocutuzumab had tumor shrinkage (*n* = 5/6 [83%]). Three patients (*n* = 3/6 [50%]) had a partial response per RECIST, version 1.1, criteria, and 3 (*n* = 3/6 [50%]) had a best response of stable disease. Patients who received zenocutuzumab had a median of 1.5 lines of prior systemic therapy (range = 0-5). Median PFS was 11 months (95% CI = 4.9 to not reached) (Figure S3A), and median time to treatment discontinuation was 10.5 months (95% CI = 5.0 to not reached) (Figure S3B). One patient developed asymptomatic radiographic progression 12.8 months into treatment and underwent radiation to oligoprogressive metastasis. They continued zenocutuzumab at the data cutoff, 45.3 months into treatment. Four of the 6 patients had elevated cancer antigen 19-9 at baseline (values: <1, 5, 123, 262, 337, and 418 U/mL; normal range = 0-40 U/mL), and 1 of the 2 patients with a baseline carcinoembryonic antigen level available had an elevated level (1.5 and 32 ng/mL; normal range = 0-5 ng/mL). All 5 patients with an elevated tumor marker at baseline had a tumor marker decline greater than 50% on treatment. One patient was treated with a HER3-targeted monoclonal antibody after disease progression on zenocutuzumab and experienced primary progression. Another patient received afatinib after progression on zenocutuzumab but discontinued therapy after 2 weeks due to poor tolerability.

## Discussion

*NRG1* fusions are a newly described, clinically actionable molecular target in solid tumors. Despite the recent accelerated Food and Drug Administration approval of zenocutuzumab in *NRG1* fusion-positive PDAC and NSCLC, little is known about the optimal detection of as well as clinical and pathological characteristics of patients with *NRG1* fusions. This study is the largest to describe *NRG1* fusion-positive patients across solid tumor histologies, identifies challenges in *NRG1* fusion detection, and is the first to describe the unique pathology in *NRG1* fusion-positive PDAC.

*NRG1* is a large gene with multiple isoforms, several transcription start sites, alternative splicing, and tissue-specific expression.^31-34^ These features add complexity to fusion detection. The broad introns are too large to tile on tissue or plasma DNA-based panel next-generation sequencing assays used commonly for cancer genotyping. In select cases, however, *NRG1* fusions can be detected on DNA-based assays when the fusion partner is on the panel. We identified 14 *NRG1* fusions on MSK-IMPACT testing, in all cases due to the upstream partner being tiled by the assay. Notably, 12 patients with NSCLC had detectable fusions on DNA, but no patient with PDAC was identified using DNA. This discrepancy is due to the design of MSK-IMPACT and other next-generation sequencing panels, which deliberately include genes such as *CD74* that are common shared fusion partners among oncogenic lung cancer fusions (*ALK, ROS1, RET, TRK*). In contrast, targetable fusions in PDAC are rare, and next-generation sequencing panels have not been tailored to detect PDAC fusion partners.^35^ In our cohort, the most common fusion partner in PDAC was *ATP1B1*, which occurred in half of the cases. *ATP1B1* has rarely been described as a fusion partner with other genes in pancreatic and gastric cancer.^36^^,^^37^ RNA-based testing is not affected by intronic size and is therefore superior for *NRG1* fusion detection. Although this is particularly true for patients with non-NSCLC disease and patients with novel fusion partners, RNA can also identify NSCLC fusions with common partners that DNA-based testing misses due to more complex structural rearrangements.^25^ For reliable *NRG1* fusion detection using targeted RNA sequencing, *NRG1* must be included on the assay panel. Panel-based RNA sequencing using hybrid capture or amplicon-based methods, such as MSK-Fusion, identifies gene fusions in a preselected panel and requires that specific primers or baits be included for the gene of interest or fusion partner. In contrast, whole transcriptome sequencing uses an unbiased approach that evaluates all expressed fusion transcripts (eliminating the requirement for specific *NRG1* primers or baits), often at the expense of increased cost, computational requirements, and turn-around time compared with targeted assays. Importantly, no single *NRG1* isoform contains all *NRG1* exons to use as a universal reference for RNA sequencing. Using multiple references may increase the yield of RNA testing. It is critical that clinicians understand the limitations of the molecular testing they use in practice. To increase sensitivity, RNA testing using a targeted panel that includes *NRG1* or *WTS* should be considered in any patient who appears to have driver-negative cancer on DNA next-generation sequencing, particularly for mitogen-activated protein kinase–driven tumors, as in lung and pancreatic cancers. To increase specificity, RNA testing should also be considered in cases with *NRG1* structural variants of unknown significance on DNA-based next-generation sequencing to determine whether the DNA-level rearrangement is transcribed into a pathogenic fusion or represents a nonfunctional genomic structural variant. Increasingly, combined DNA/RNA panels and more comprehensive assays such as whole transcriptome sequencing are becoming commercially available that may overcome some of these challenges.

In our cohort, the most common *NRG1* fusion-positive tumor types were NSCLC (60%) and PDAC (21%), followed by breast (10%) and hepatobiliary (6%) cancers. This finding is consistent with what was observed on the pivotal eNRGy clinical trial.^16^ Notably, there were patients with other tumor types, including colorectal cancer, sarcoma, salivary gland cancer, and prostate cancer, who had structural variants of unknown significance upon detailed review. It is therefore critical to confirm that *NRG1* fusions are likely oncogenic by expert review or RNA-based next-generation sequencing, particularly when the fusion partner or cancer type is not well described in *NRG1* fusion-positive cancer.^6^^,^^16^ In the eNRGy study, responses to zenocutuzumab were observed in patients with breast, hepatobiliary, and other solid tumor types. Additional clinical data are necessary to determine whether the clear benefit of zenocutuzumab observed in NSCLC and PDAC extends to other cancer types.

PDAC is an aggressive disease with limited therapeutic options. The majority of cases (>90%) are driven by variations in *KRAS*^38^; less is known about *KRAS* wild-type PDAC. In this cohort of 10 patients with *NRG1* fusion-positive PDAC, the largest to date, we describe the genomic, clinical, and pathologic features of *NRG1* fusion-positive PDAC and clinical outcomes on currently available therapy. *NRG1* fusions occurred exclusively in patients with *KRAS* wild-type disease, in both sexes, regardless of smoking history. They also occurred in younger patients: The median age at diagnosis was 48.5 years of age compared with the general population, where most cases are diagnosed in patients older than 65 years.^39^ This finding is consistent with prior reports demonstrating earlier onset in *KRAS* wild-type vs mutated PDAC and demographic data from patients with *NRG1* fusion-positive PDAC enrolled in the phase 2 eNRGy study.^8^^,^^16^^,^^40^ Pathological review in our *NRG1* fusion-positive cohort demonstrated adenocarcinoma with prominent cellularity, reduced intracytoplasmic mucin, and a sparse stromal component in contrast with conventional PDAC with abundant intracytoplasmic mucin and stroma. These unique clinical (early-onset) and pathological features, in addition to *KRAS* wild-type status on DNA testing, should prompt genetic testing to look for *NRG1* fusions in clinical practice.

The median real-world PFS on first-line chemotherapy was 12.6 months in our study, which is numerically longer than reported in clinical trials of standard upfront chemotherapy for PDAC with FOLFIRINOX or gemcitabine plus nab-paclitaxel (6.4 and 5.5 months, respectively).^41^^,^^42^ Due to the relatively small sample size in our cohort, the confidence intervals are wide, and larger studies are needed to understand whether *NRG1* fusion-positive PDAC has unique predictive or prognostic implications on standard therapy. *NRG1* fusions do predict response to zenocutuzumab, with an impressive overall response rate of 50% (3/6) and tumor shrinkage with tumor marker decline in most patients. The median PFS was 11 months, despite 5 of 6 patients receiving zenocutuzumab as second-line to sixth-line therapy. In contrast, the overall response rate to standard second-line chemotherapy with liposomal irinotecan, fluorouracil, and leucovorin was 16%, with a median PFS of only 3.1 months.^43^ In our cohort, the median overall survival from diagnosis of metastatic disease was 37.6 months, which is considerably longer than that reported in PDAC clinical trials (8.5-11.1 months).^41^^,^^42^ This discrepancy may be due to the differences in biology; natural variability, given the small sample size; differences in definition (survival from diagnosis vs treatment start); or improved outcomes with use of zenocutuzumab. Importantly, Varghese et al.^40^ reported improved overall survival in *KRAS* wild-type vs mutated PDAC, consistent with *KRAS* wild-type cancers having a distinct biology.

The limitations of this study include its small sample size and selection bias from single-institution data. Additional single-institution or multicenter studies are needed to validate our findings. Nonetheless, this is the largest reported cohort of *NRG1* fusion-positive pancreatic cancer using real-world data.

In conclusion, *NRG1* fusions are a newly described, clinically actionable target in solid tumors. *NRG1* fusion-positive PDAC has a unique histology compared with conventional PDAC and predicts response to HER2-/HER3-targeted therapy. RNA-based genomic testing is critical to identify patients with *NRG1* fusion-positive disease in clinical practice.

## Supplementary Material

djaf361_Supplementary_Data

## Data Availability

Data that support the findings of this study are available upon reasonable request from the corresponding author.

## References

[1] Nikanjam M , Okamura R, Barkauskas DA, Kurzrock R. Targeting fusions for improved outcomes in oncology treatment. Cancer. 2020;126:1315-1321. 31794076 10.1002/cncr.32649 PMC7050395

[2] Schram AM , Chang MT, Jonsson P, Drilon A. Fusions in solid tumours: diagnostic strategies, targeted therapy, and acquired resistance. Nat Rev Clin Oncol. 2017;14:735-748. 28857077 10.1038/nrclinonc.2017.127 PMC10452928

[3] Tzahar E , Levkowitz G, Karunagaran D, et al ErbB-3 and ErbB-4 function as the respective low and high affinity receptors of all Neu differentiation factor/heregulin isoforms. J Biol Chem. 1994;269:25226-25233. 7929212

[4] Pinkas-Kramarski R , Shelly M, Guarino BC, et al ErbB tyrosine kinases and the two neuregulin families constitute a ligand-receptor network. Mol Cell Biol. 1998;18:6090-6101. 9742126 10.1128/mcb.18.10.6090 PMC109195

[5] Yarden Y , Sliwkowski MX. Untangling the ErbB signalling network. Nat Rev Mol Cell Biol. 2001;2:127-137. 11252954 10.1038/35052073

[6] Jonna S , Feldman RA, Swensen J, et al Detection of NRG1 gene fusions in solid tumors. Clin Cancer Res. 2019;25:4966-4972. 30988082 10.1158/1078-0432.CCR-19-0160 PMC7470623

[7] Drilon A , Somwar R, Mangatt BP, et al Response to ERBB3-directed targeted therapy in NRG1-rearranged cancers. Cancer Discov. 2018;8:686-695. 29610121 10.1158/2159-8290.CD-17-1004 PMC5984717

[8] Heining C , Horak P, Uhrig S, et al NRG1 fusions in KRAS wild-type pancreatic cancer. Cancer Discov. 2018;8:1087-1095. 29802158 10.1158/2159-8290.CD-18-0036

[9] Chang JC , Offin M, Falcon C, et al Comprehensive molecular and clinicopathologic analysis of 200 pulmonary invasive mucinous adenocarcinomas identifies distinct characteristics of molecular subtypes. Clin Cancer Res. 2021;27:4066-4076. 33947695 10.1158/1078-0432.CCR-21-0423 PMC8282731

[10] Drilon A , Duruisseaux M, Han JY, et al Clinicopathologic features and response to therapy of NRG1 fusion-driven lung cancers: the eNRGy1 global multicenter registry. J Clin Oncol. 2021;39:2791-2802. 34077268 10.1200/JCO.20.03307 PMC8407651

[11] Fernandez-Cuesta L , Plenker D, Osada H, et al CD74-NRG1 fusions in lung adenocarcinoma. Cancer Discov. 2014;4:415-422. 24469108 10.1158/2159-8290.CD-13-0633

[12] Jung Y , Yong S, Kim P, et al VAMP2-NRG1 fusion gene is a novel oncogenic driver of non-small-cell lung adenocarcinoma. J Thorac Oncol. 2015;10:1107-1111. 26134228 10.1097/JTO.0000000000000544

[13] Shin DH , Lee D, Hong DW, et al Oncogenic function and clinical implications of SLC3A2-NRG1 fusion in invasive mucinous adenocarcinoma of the lung. Oncotarget. 2016;7:69450-69465. 27626312 10.18632/oncotarget.11913 PMC5342490

[14] Fernandez-Cuesta L , Thomas RK. Molecular pathways: targeting NRG1 fusions in lung cancer. Clin Cancer Res. 2015;21:1989-1994. 25501131 10.1158/1078-0432.CCR-14-0854

[15] Werr L , Plenker D, Dammert MA, et al CD74-NRG1 fusions are oncogenic in vivo and induce therapeutically tractable ERBB2: ERBB3 heterodimerization. Mol Cancer Ther. 2022;21:821-830. 35247925 10.1158/1535-7163.MCT-21-0820 PMC9377738

[16] Schram AM , Goto K, Kim DW, et al; eNRGy Investigators. Efficacy of zenocutuzumab in NRG1 fusion-positive cancer. N Engl J Med. 2025;392:566-576. 39908431 10.1056/NEJMoa2405008 PMC11878197

[17] Carrizosa DR , Burkard ME, Elamin YY, et al CRESTONE: initial efficacy and safety of seribantumab in solid tumors harboring NRG1 fusions. J Clin Oncol. 2022;40:3006-3006.35786967

[18] Liu SV , Frohn C, Minasi L, et al Real-world outcomes associated with afatinib use in patients with solid tumors harboring NRG1 gene fusions. Lung Cancer. 2024;188:107469. 38219288 10.1016/j.lungcan.2024.107469

[19] Eubank MH , Hyman DM, Kanakamedala AD, Gardos SM, Wills JM, Stetson PD. Automated eligibility screening and monitoring for genotype-driven precision oncology trials. J Am Med Inform Assoc. 2016;23:777-781. 27016727 10.1093/jamia/ocw020 PMC6370254

[20] Cheng DT , Mitchell TN, Zehir A, et al Memorial Sloan Kettering-Integrated Mutation Profiling of Actionable Cancer Targets (MSK-IMPACT): a hybridization capture-based next-generation sequencing clinical assay for solid tumor molecular oncology. J Mol Diagn. 2015;17:251-264. 25801821 10.1016/j.jmoldx.2014.12.006 PMC5808190

[21] Middha S , Zhang L, Nafa K, et al Reliable pan-cancer microsatellite instability assessment by using targeted next-generation sequencing data. J Clin Oncol Precis Oncol. 2017;2017. 10.1200/PO.17.00084 PMC6130812 30211344

[22] Shen R , Seshan VE. FACETS: allele-specific copy number and clonal heterogeneity analysis tool for high-throughput DNA sequencing. Nucleic Acids Res. 2016;44:e131. 27270079 10.1093/nar/gkw520 PMC5027494

[23] Zheng Z , Liebers M, Zhelyazkova B, et al Anchored multiplex PCR for targeted next-generation sequencing. Nat Med. 2014;20:1479-1484. 25384085 10.1038/nm.3729

[24] Zehir A , Benayed R, Shah RH, et al Mutational landscape of metastatic cancer revealed from prospective clinical sequencing of 10,000 patients. Nat Med. 2017;23:703-713. 28481359 10.1038/nm.4333 PMC5461196

[25] Benayed R , Offin M, Mullaney K, et al High Yield of RNA sequencing for targetable kinase fusions in lung adenocarcinomas with no mitogenic driver alteration detected by DNA sequencing and low tumor mutation burden. Clin Cancer Res. 2019;25:4712-4722. 31028088 10.1158/1078-0432.CCR-19-0225 PMC6679790

[26] Kehl KL , Riely GJ, Lepisto EM, et al; American Association of Cancer Research (AACR) Project Genomics Evidence Neoplasia Information Exchange (GENIE) Consortium. Correlation between surrogate end points and overall survival in a multi-institutional clinicogenomic cohort of patients with non-small cell lung or colorectal cancer. JAMA Netw Open. 2021;4:e2117547. 34309669 10.1001/jamanetworkopen.2021.17547 PMC8314138

[27] Lauinger M , Christen D, Klar RFU, et al BRAF(Δβ3-αC) in-frame deletion mutants differ in their dimerization propensity, HSP90 dependence, and druggability. Sci Adv. 2023;9:eade7486. 37656784 10.1126/sciadv.ade7486 PMC11804575

[28] Basturk O , Adsay V, Askan G, et al Intraductal tubulopapillary neoplasm of the pancreas: a clinicopathologic and immunohistochemical analysis of 33 cases. Am J Surg Pathol. 2017;41:313-325. 27984235 10.1097/PAS.0000000000000782 PMC5309137

[29] Basturk O , Berger MF, Yamaguchi H, et al Pancreatic intraductal tubulopapillary neoplasm is genetically distinct from intraductal papillary mucinous neoplasm and ductal adenocarcinoma. Mod Pathol. 2017;30:1760-1772. 28776573 10.1038/modpathol.2017.60

[30] Paolino G , Esposito I, Hong SM, et al Intraductal tubulopapillary neoplasm (ITPN) of the pancreas: a distinct entity among pancreatic tumors. Histopathology. 2022;81:297-309. 35583805 10.1111/his.14698 PMC9544156

[31] Nagasaka M , Ou SI. NRG1 and NRG2 fusion positive solid tumor malignancies: a paradigm of ligand-fusion oncogenesis. Trends Cancer. 2022;8:242-258. 34996744 10.1016/j.trecan.2021.11.003

[32] Steinthorsdottir V , Stefansson H, Ghosh S, et al Multiple novel transcription initiation sites for NRG1. Gene. 2004;342:97-105. 15527969 10.1016/j.gene.2004.07.029

[33] Falls DL. Neuregulins: functions, forms, and signaling strategies. Exp Cell Res. 2003;284:14-30. 12648463 10.1016/s0014-4827(02)00102-7

[34] Meyer D , Yamaai T, Garratt A, et al Isoform-specific expression and function of neuregulin. Development. 1997;124:3575-3586. 9342050 10.1242/dev.124.18.3575

[35] Gkountakos A , Singhi AD, Westphalen CB, Scarpa A, Luchini C. Fusion genes in pancreatic tumors. Trends Cancer. 2024;10:430-443. 38378317 10.1016/j.trecan.2024.01.009 PMC13526370

[36] Shinozaki-Ushiku A , Ishikawa S, Komura D, Seto Y, Aburatani H, Ushiku T. The first case of gastric carcinoma with NTRK rearrangement: identification of a novel ATP1B-NTRK1 fusion. Gastric Cancer. 2020;23:944-947. 32189226 10.1007/s10120-020-01061-9

[37] Lee NK. Squamous cell carcinoma of the pancreas with a pancreatic intraductal papillary mucinous neoplasm: a case report. KMJ. 2024;39:71-74.

[38] Biankin AV , Waddell N, Kassahn KS, et al; Australian Pancreatic Cancer Genome Initiative. Pancreatic cancer genomes reveal aberrations in axon guidance pathway genes. Nature. 2012;491:399-405. 23103869 10.1038/nature11547 PMC3530898

[39] GBD 2017 Pancreatic Cancer Collaborators. The global, regional, and national burden of pancreatic cancer and its attributable risk factors in 195 countries and territories, 1990-2017: a systematic analysis for the Global Burden of Disease Study 2017. Lancet Gastroenterol Hepatol. 2019;4:934-947. https://doi.org/10.1016/S2468-1253(19)30347-4 31648972 10.1016/S2468-1253(19)30347-4 PMC7026711

[40] Varghese AM , Perry MA, Chou JF, et al Clinicogenomic landscape of pancreatic adenocarcinoma identifies KRAS mutant dosage as prognostic of overall survival. Nat Med. 2025;31:466-477. 39753968 10.1038/s41591-024-03362-3 PMC11835752

[41] Conroy T , Desseigne F, Ychou M, et al; PRODIGE Intergroup. FOLFIRINOX versus gemcitabine for metastatic pancreatic cancer. N Engl J Med. 2011;364:1817-1825. 21561347 10.1056/NEJMoa1011923

[42] Von Hoff DD , Ervin T, Arena FP, et al Increased survival in pancreatic cancer with nab-paclitaxel plus gemcitabine. N Engl J Med. 2013;369:1691-1703. 24131140 10.1056/NEJMoa1304369 PMC4631139

[43] Wang-Gillam A , Li CP, Bodoky G, et al; NAPOLI-1 Study Group. Nanoliposomal irinotecan with fluorouracil and folinic acid in metastatic pancreatic cancer after previous gemcitabine-based therapy (NAPOLI-1): a global, randomised, open-label, phase 3 trial. Lancet. 2016;387:545-557. 26615328 10.1016/S0140-6736(15)00986-1

