# Supplementary material for: NRG1 fusion-positive solid tumors: clinical detection, genomic landscape, and real-world data in pancreatic cancer
Source: J Natl Cancer Inst. 2025 Dec 13;118(5):847–55. doi: 10.1093/jnci/djaf361 (PMC13155229; doi:10.1093/jnci/djaf361)
Supplement: djaf361_Supplementary_Data [file djaf361_supplementary_data.zip › JNCI_Supplementary Figure legends.docx]

**Supplementary Material**

**Figure S1.** Radiographic representation of NRG1+ PDAC with pancreatic body infiltrative tumor encasing the superior mesenteric artery (arrowhead) and with bilobar hypovascular liver metastases (arrows).

**Figure S2.** Clinical outcomes of patients with NRG1+ PDAC. Kaplan-Meier Survival plots showing (**A**) progression-free survival (PFS) on first-line chemotherapy (**B**) time to treatment discontinuation (**C**) overall survival (OS) from diagnosis, and (**D**) overall survival from the diagnosis of metastatic disease.

**Figure S3.** Clinical outcomes of patients with NRG1+ PDAC on HER2/HER3-targeted therapy. Kaplan-Meier Survival plots showing (**A**) progression-free survival on HER2/HER3-targeted therapy (zenocutuzumab), and (**B**) time to treatment discontinuation with HER2/HER3-targeted therapy (zenocutuzumab).
