# Supplementary figures and images for: NRG1 fusion-positive solid tumors: clinical detection, genomic landscape, and real-world data in pancreatic cancer
Source: J Natl Cancer Inst. 2025 Dec 13;118(5):847–55. doi: 10.1093/jnci/djaf361 (PMC13155229; doi:10.1093/jnci/djaf361)

**Supplementary Material**  
Figure S1

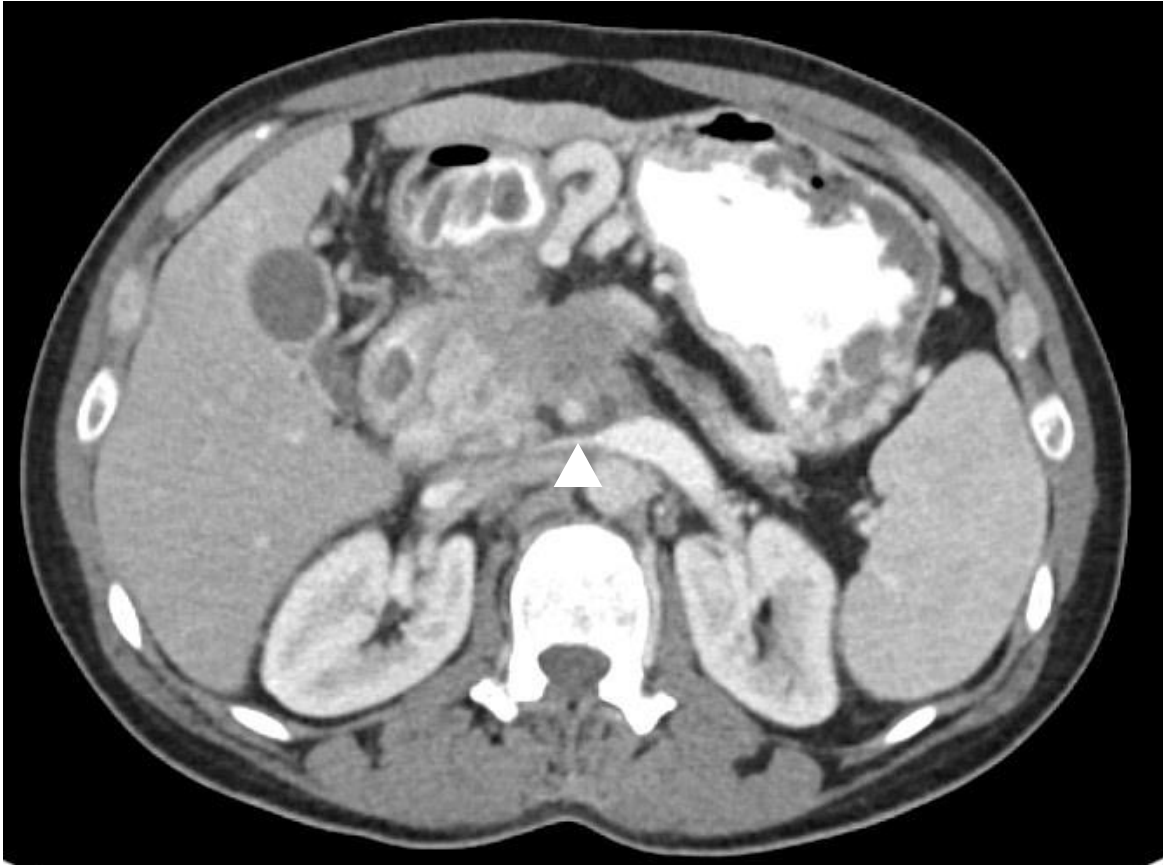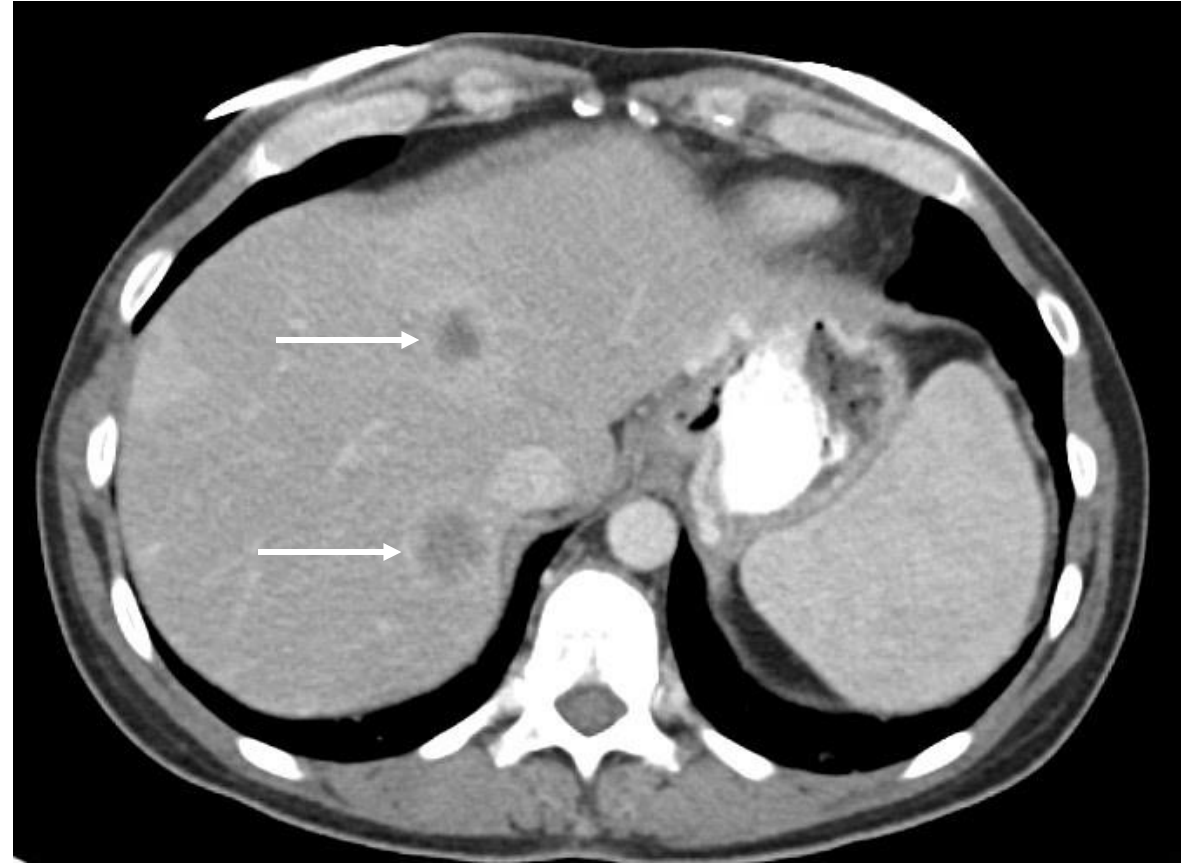

Supplement: djaf361_Supplementary_Data [file djaf361_supplementary_data.zip › Figure S1.pdf]

Supplementary Material

Figure S3

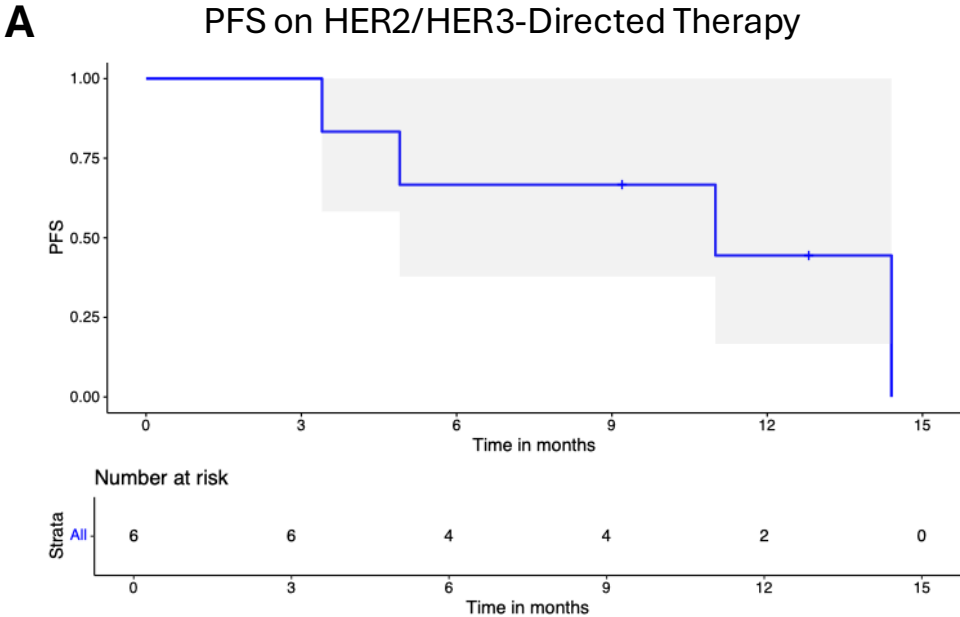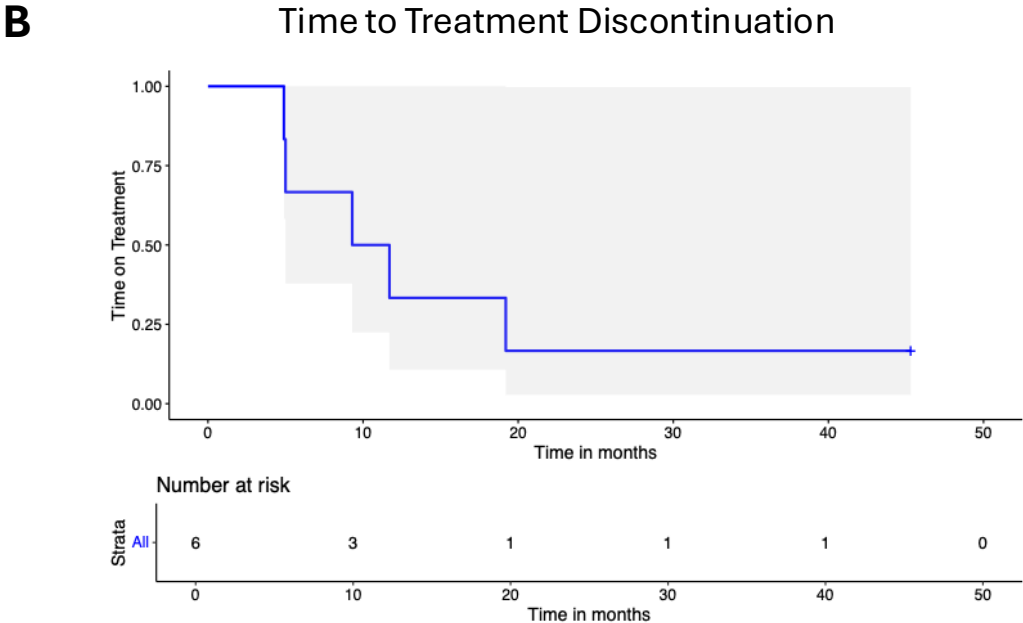

Supplement: djaf361_Supplementary_Data [file djaf361_supplementary_data.zip › Figure S3.pdf]
